# A gene expression profile for the lower osteogenic potent of bone-derived MSCs from osteoporosis with T2DM and the potential mechanism

**DOI:** 10.1186/s13018-022-03291-2

**Published:** 2022-09-01

**Authors:** Sheng-li Xia, Zi-yuan Ma, Bin Wang, Feng Gao, Sheng-yang Guo, Xu-han Chen

**Affiliations:** 1 Department of Orthopedics, Shanghai University of Medicine and Health Sciences Affiliated Zhoupu Hospital, Shanghai, 201318 China; 2 Zhoupu Community Health Service Center, 163 Shenmei East Road, Pudong New Area, Shanghai, 201318 China

**Keywords:** T2DM, Osteoporosis, FOXQ1, Osteogenic differentiation, Mesenchymal stem cells

## Abstract

**Background:**

Osteoporosis (OP) patients complicated with type II diabetes mellitus (T2DM) has a higher fracture risk than the non-diabetic patients, and mesenchymal stem cells (MSCs) from T2DM patients also show a weaker osteogenic potent. The present study aimed to provide a gene expression profile in MSCs from diabetic OP and investigated the potential mechanism.

**Methods:**

The bone-derived MSC (BMSC) was isolated from OP patients complicated with or without T2DM (CON-BMSC, T2DM-BMSC). Osteogenic differentiation was evaluated by qPCR analysis of the expression levels of osteogenic markers, ALP activity and mineralization level. The differentially expressed genes (DEGs) in T2DM-BMSC was identified by RNA-sequence, and the biological roles of DEGs was annotated by bioinformatics analyses. The role of silencing the transcription factor (TF), Forkhead box Q1 (FOXQ1), on the osteogenic differentiation of BMSC was also investigated.

**Results:**

T2DM-BMSC showed a significantly reduced osteogenic potent compare to the CON-BMSC. A total of 448 DEGs was screened in T2DM-BMSC, and bioinformatics analyses showed that many TFs and the target genes were enriched in various OP- and diabetes-related biological processes and pathways. FOXQ1 had the highest verified fold change (abs) among the top 8 TFs, and silence of FOXQ1 inhibited the osteogenic differentiation of CON-BMSC.

**Conclusions:**

Our study provided a comprehensive gene expression profile of BMSC in diabetic OP, and found that downregulated FOXQ1 was responsible for the reduced osteogenic potent of T2DM-BSMC. This is of great importance for the special mechanism researches and the treatment of diabetic OP.

**Supplementary Information:**

The online version contains supplementary material available at 10.1186/s13018-022-03291-2.

## Introduction

Osteoporosis (OP) is a common degenerative disease worldwide, and characterized by micro-architectural deterioration of bone tissue, loss of bone mass and a propensity to fracture [1]. It is reported that OP affects more than 30% of the adults (over the age of 50 years) in China [2], and the economic burden of OP-related fracture reaches approximately $17.9 billion per annum in the USA [3]. Type 2 diabetes mellitus (T2DM), accounting for the 90% of all the diabetes mellitus cases, is considered to be a risk factor for osteoporosis [4], and the pooled prevalence rate of OP in T2DM patients reaches ~ 40% [5]. In addition, cumulative 10-year incidence of fractures in newly diagnosed T2DM patients is above 30%, significantly higher than the non-diabetic controls [6]. T2DM is characterized by insulin resistance or the dysfunction of pancreatic islet β-cells, resulting in hyperglycemia and hyperinsulinemia [7]. This sustained abnormal blood internal environment often causes the imbalance of bone metabolism, characterized with reduced osteoblast maturation, increased osteoclastic activity, and finally lead to marrow adipogenesis, bone loss and increased fracture risk [8].

Mesenchymal stem cells (MSCs) are a breed of undifferentiated cells with self-proliferation ability and can differentiate into multiple cell lineages, including osteoblasts, adipocytes, fibroblasts, and chondrocytes [9]. Transplantation of MSC to increase osteoblast differentiation and to rebalance bone metabolism has been regarded as a promising treatment for OP. Many studies have proved the positive therapeutic outcomes after transplantation of autologous or allogeneic MSC through intra-bone marrow or intra-tail venous. However, MSC transplantation for diabetic OP is less investigated, and also transplanted MSCs used for patients complicated with DM is not satisfied [10, 11]. One major reason is the biological characteristics of MSCs is altered in the diabetic micro-environment [12]. For example, co-culture of T2DM serum and MSCs results in the inhibition of osteogenic differentiation of MSCs [13]. Additionally, autologous marrow MSCs of T2DM patients also show preferential differentiation toward adipocytes, other than the osteoblasts [14, 15]. Therefore, understanding of the molecular alterations of MSCs in diabetic micro-environment is of great importance to enhance the therapeutic effect of MSCs transplantation.

In the present study, bone-derived mesenchymal stem cells (BMSC) from OP patients complicated with or without T2DM was isolated (CON-BMSC, T2DM-BMSC) and the osteogenic potent of the two was compared. RNA-sequence was used to identify differentially expressed genes (DEGs) in T2DM-BMSC, and the biological roles of the DEGs was annotated by bioinformatics analyses. Furthermore, transcription factors (TFs) from the DEGs were also screened out, and one of the TFs, forkhead box Q1 (FOXQ1), was proved to be responsible for the inhibited osteogenic potent of T2DM-BMSC.

## Materials and methods

### Specimens

Patients undergoing hip replacement surgery were included in this study. OP Patients with a diabetic history more than 10 years were included in the T2DM group, while those without diabetic history were included in the control (CON) group. Eight patients were recruited in each group. The exclusive criteria: femoral necrosis, congenital hip dysplasia, disorders that affect the bone, a history of using drugs regulating bone metabolism, a history of tumor. OP was diagnosed by X-rays, and bone mineral density (BMD) was used to evaluate the degree of OP. Demographic characteristics of the 16 patients in the two groups is presented in Table 1. The study was approved by the Ethics Committee of the Zhoupu Hospital, Shanghai University of Medicine & Health Sciences, Shanghai, China, and informed consent was signed by all the patients. All the fresh bone specimens were harvested under sterile conditions and transferred to the laboratory as soon as possible for cell isolation.

**Table 1:** Demographic characteristics of the patients

Items	CON	T2DM	*p* Value
Age (years)	76.13 ± 4.42	74.38 ± 5.10	0.475
Sex	F(4), M(4)	F(5), M(3)	–
BMI (kg/m^2^)	22.53 ± 1.27	23.43 ± 0.81	0.113
T-scores	− 3.21 ± 0.34	− 3.31 ± 0.31	0.545

*F* female, *M* male, *BMI* body mass indices

### Cell isolation and culture

Primary BMSCs were isolated from the bone specimens following the previous study [16]. Breifly, the specimens were digested in 1 mg/mL collagenase II (Gibco) at 37 °C for 3 h. The digested tissue suspension was filtered, and the obtained cells were cultured in MEM medium added with 10% fetal bovine serum, 1% streptomycin and 1% penicillin (all Gibco). All the cell cultures were maintained at 37 °C in a 5% humidified CO_2_ atmosphere. BMSC isolated from CON and BMSC groups were named as CON-BMSC and T2DM-BMSC, respectively.

### Osteogenic induction

Cells were seeded in a six-well plate at 2 × 10^5^ cells/well. The complete MEM medium supplied with 0.1 mg/ml dexamethasone (Sigma-Aldrich, USA), 50 g/ml of ascorbic acid (Sigma-Aldrich, USA), and 10 mM glycerophosphate (Sigma-Aldrich, USA) was used to induce osteogenic differentiation for 7 or 21 days. The medium was exchanged every 3 days.

### Quantitative polymerase chain reaction (qPCR)

After cells experienced an 7-day osteogenic induction or transfected the purpose sequences, the total RNA was extracted using Trizol reagent (Ambion, Life Technologies, Germany) and chloroform/isoamyl alcohol (24:1) (PanReac AppliChem, Germany). A cDNA Synthesis Kit (Roche Diagnostics GmbH, Germany) was used to reverse transcribe 1 µg mRNA following the provided protocols. Next, qPCR was done using LightCycler 480 SYBR Green I Master (Roche Diagnostics, GmbH) according to the manufacturer's instructions. The amplification program was run at 95 °C for 10 s, 60 °C for 20 s, and 72 °C for 20 s for 45 cycles. The various primers sequences used is listed in Table 2. The data were normalized to the GAPDH expression level and presented as the average from three experiments. The relative expression was calculated using the 2^−ΔΔCt^ method.

**Table 2:** Primer sequences used for qPCR

Genes	Forward (5′–3′)	Reverse (5′–3′)
RUNX2	TGGTTACTGTCATGGCGGGTA	TCTCAGATCGTTGAACCTTGCTA
COL1A1	GAGGGCCAAGACGAAGACATC	CAGATCACGTCATCGCACAAC
OPN	CTCCATTGACTCGAACGACTC	CAGGTCTGCGAAACTTCTTAGAT
SPI1	GTGCCCTATGACACGGATCTA	AGTCCCAGTAATGGTCGCTAT
RBAK	AAGCTATGCTAGGACAAAACCTG	GCTTCTCCCCTATGTAAGCTCTC
PLEK	AAGAAGGGGAGCGTGTTCAAT	TCAGCGGGATCATTCCTTTGG
DLX3	TACCCTGCCCGAGTCTTCTG	TGGTGGTAGGTGTAGGGGTTC
FERD3L	GCTGGACTTCGTCGCAGAC	GCCTAATAGGGAGACACCTCTTC
FOXQ1	CACGCAGCAAGCCATATACG	CGTTGAGCGAAAGGTTGTGG
BARX1	TTCCACGCCGGACAGAATAGA	AGTAAGCTGCTCGCTCGTTG
PITX1	GTTCAGCGGCCTAGTGCAG	CGGGCTCATGGAGTTGAAGAA
GAPDH	GGAGCGAGATCCCTCCAAAAT	GGCTGTTGTCATACTTCTCATGG

### Alkaline phosphatase (ALP) staining and activity detection

After cells were incubated with osteogenic medium for 7 days, 4% paraformaldehyde (Carl Roth GmbH, Karlsruhe, Germany) was added to the wells for a fixing of 20 min. Next, cells were incubated with BCIP/NBT stain (Beyotime, Shanghai, China) for another 1 h in the dark, and a conventional camera was used to record the staining result. For the detection of ALP activity, cells were lysed after induction for 7 days, and an ALP detection kit (Beyotime) was used to detect the activity following manufacturer’s instructions.

### Alizarin Red S staining (ARS staining)

Cells were fixed with 4% paraformaldehyde (Carl Roth GmbH) and stained with 40 mM ARS reagent (Sigma–Aldrich, Darmstadt, Germany) after osteogenic induction for 21 days. Images of the calcified matrices were photographed using a conventional camera. For quantification, the calcified matrices was resolved using 10% cetylpyridinium chloride for 1 h, and spectrophotometric quantification was performed at 562 nm.

### RNA-sequence (RNA-seq)

BMSC from the two groups (*n* = 3) was randomly selected and used for RNA-seq. Briefly, a RNeasy mini kit (Qiagen, Germany) was used to isolate the total RNA. According to the manufacturer’s guidelines, to synthesize paired-end libraries, we used TruSeq™ RNA sample preparation kit (Illumina, USA). Magnetic beads attached with Poly-T oligo were used to purify the poly-A containing mRNA molecules. mRNA fragmented into small pieces using divalent cations under 94℃ for 8 min. The cleaved RNA fragments are used to convert it to cDNA using reverse transcriptase and random primers, followed by cDNA synthesis using DNA polymerase I and RNase H. PCR was done to create the final cDNA library. Qubit^®^ 2.0 Fluorimeter (Life Technologies, USA) was used to quantify purified libraries and validated by Agilent 2100 bioanalyzer (Agilent Technologies, USA). Construction of library and sequencing were performed by Sinotech Genomics Co., Ltd (Shanghai, China). We used R package edgeR to analyze the differential expression of mRNA. Genes with |fold change (FC)| value > 1.5 and *p* value < 0.05 were considered as differentially expressed genes (DEGs). The screen of all the TFs from the DEGs were performed by the technician in Sinotech Genomics (shanghai, China).

### Bioinformatics analyses

Gene ontology (GO) analysis for biological processes, cellular components, molecular function, and a KEGG (Kyoto Encyclopedia of Genes and Genomes http://www.genome.ad.jp/kegg) pathway analysis via enriching R package was performed. The terms or pathways were ranked in a descending order according to the enrichment factor, and the top 30 terms or pathways were selected for visualization, respectively.

### Construction of transcriptional regulatory network

Transcription factor binding sites (TFBSs) of the DEGs was analyzed using TFBSTools [17], and the transcriptional regulatory network was visualized by Cytoscape.

### Cell transfection

Cells were seeded in six-well plates (2 × 10^5^ cells/well). According to the manufacturer's instructions, when the confluency of the cells reached ~ 80%, the cells were transfected with siRNAs using Lipofectamine^®^ 2000 (Invitrogen) instructions. After 48 h, cells were collected for qPCR analysis. Small interference RNA used for the degradation of FOXQ1 mRNA (si-FOXQ1, 5′-GCA CGC AGC AAG CCA UAU A-3′) and its negative control (si-NC, 5′-UUC UCC GAA CGU GUC ACG UTT-3′) were designed and synthesized by RiboBio Co., Ltd. (Guangzhou, China).

### Western blot analysis

Cells were lysed using RIPA buffer (Sigma-Aldrich). The BCA protein assay kit (Biosharp Life Sciences) was used to measure the total protein concentration. We used 10 µg protein and separated it by SDS-PAGE (12% gels). Additionally, the membranes were blocked in blocking buffer at room temperature for 60 min and separately incubated overnight at 4 °C with antibodies against FOXQ1 (1:1000) and GAPDH (1:1000). The next day, membranes were incubated with horseradish peroxidase (HRP)-conjugated secondary antibody (1:5000) at room temperature for 2 h. All the antibodies were purchased from ABclonal (Wuhan, China), and GAPDH was used an internal control.

### Statistical analysis

SPSS 17.0 software (SPSS Inc, Chicago, IL) was used for statistical analysis. Data are shown as the mean ± standard deviation. Unpaired Student’s *t* test was used done to analyze the differences between groups. *p* Value < 0.05 was considered as a significant difference.

## Results

### Osteogenic differentiation is inhibited in T2DM-BMSC

**Fig. 1:**
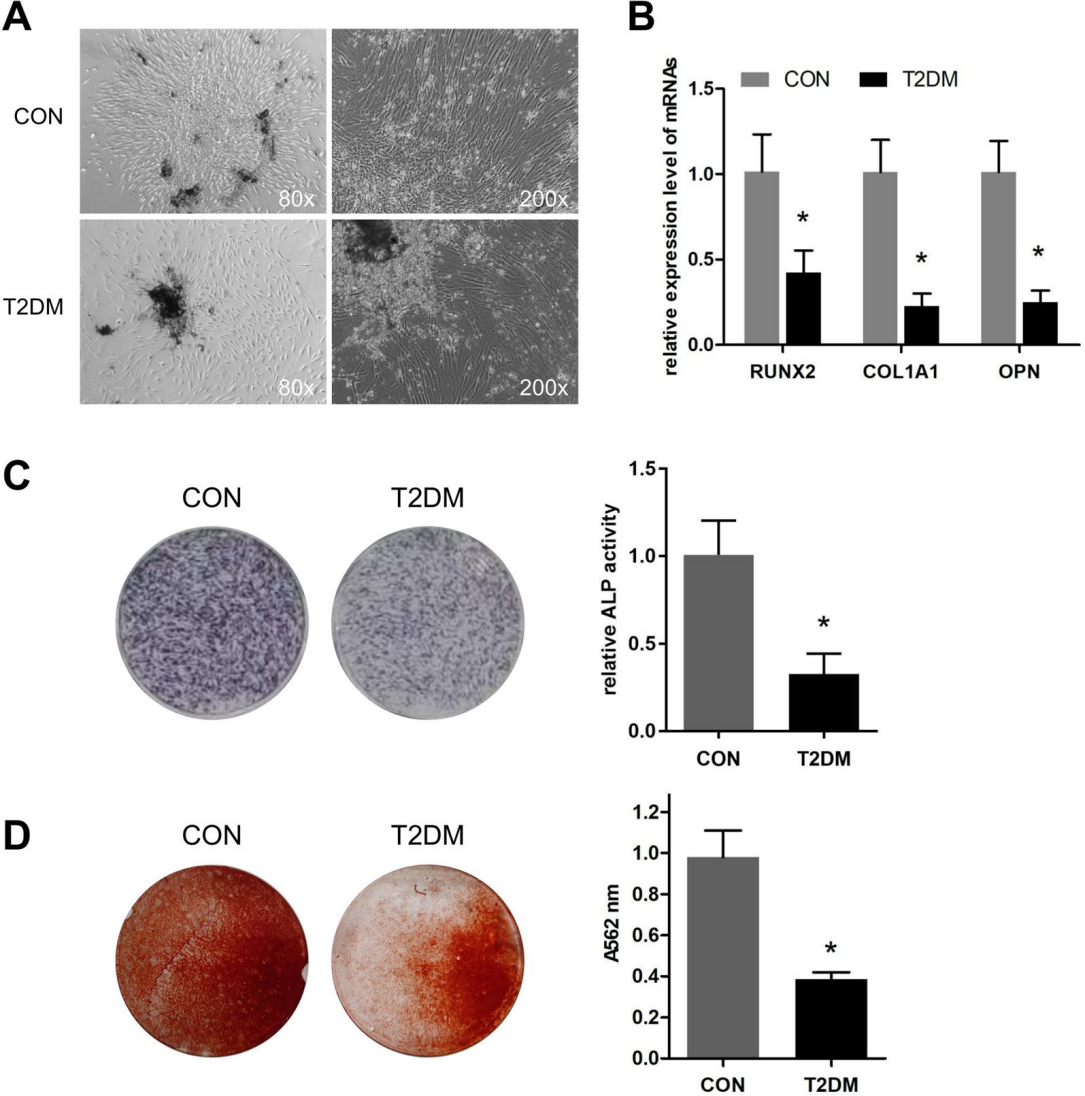
Osteogenic differentiation is inhibited in T2DM-BMSC. **A** Morphology of BMSC from CON and T2DM groups. **B** qPCR was used to detect the expression levels of three osteogenic markers after BMSC underwent a 7-day osteogenic induction. **C** ALP staining (left) and activity detection (right) were performed to detect ALP expression level of BMSC after osteogenic induction for 7 days. **D** ARS staining was used for the evaluation of mineralization level after BMSC underwent a 21-day osteogenic induction

As shown in Table 1, there was no significant difference in age or BMI or T-score of the two groups. Next, the BMSCs were isolated and observed. The morphology of the two BMSCs were both slender, and a small amount of the T2DM-BMSC had 3–6 pseudopodias (Fig. 1A). The mRNA expression of three genes (RUNX2, COL1a1, and OPN) involved in osteogenic differentiation was suppressed in T2DM group, compared to the CON group (Fig. 1B). ALP staining and activity detection results showed that ALP level in the CON group was higher than that in T2DM group (Fig. 1C). The mineralization levels was further verified by ARS staining, and the results indicated that the absorbance at A562nm induced from T2DM-BMSC was significantly reduced (Fig. 1D). Therefore, BMSC from OP patients complicated with T2DM exhibited a weaker osteogenic potent.

### RNA-seq analysis of DEGs in T2DM-BMSC

**Fig. 2:**
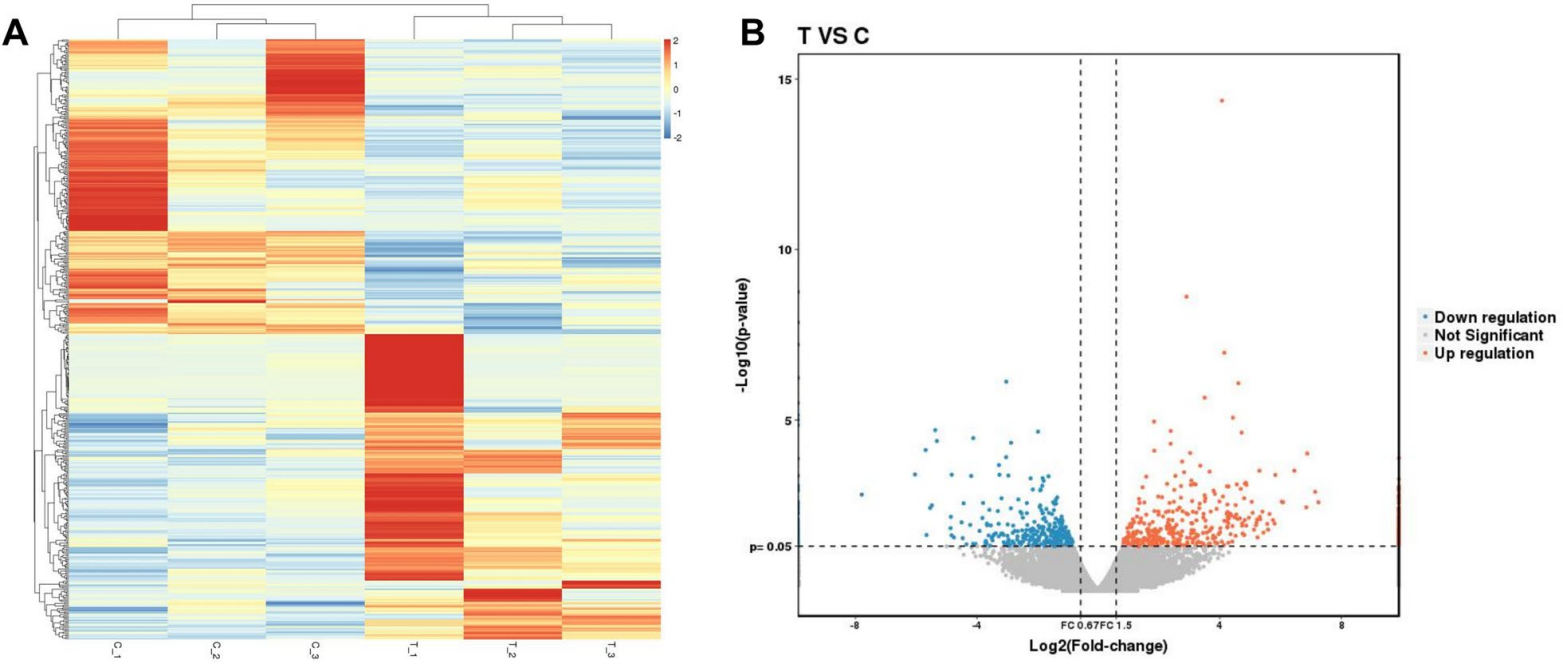
RNA-seq analysis of DEGs in T2DM-BMSC. **A** Heap-map presented the overall expression of DEGs in each samples. CON: C_1, C_2, C_3; T2DM: T_1, T_2, T_3. Each line indicates a gene, and each column indicates a sample of BMSC. **B** Volcano pot of all DEGs in T2DM group, screened under the thresholds of FC(abs) > 1.5 and *p* value < 0.05. T: T2DM; C: CON. Each dot indicate a gene. Red indicate upregulation and but indicate downregulation

DEGs between the CON and T2DM groups were screened out under the filtering conditions of FC (abs) > 1.5 and *p* value < 0.05. As a result, a total of 448 DEGs were identified in the T2DM group. The expression level of all the DEGs in each sample was presented in a clustered heat map, and the overall gene expression alteration trend of the three samples in the same group was basically consistent (Fig. 2A). A volcano plot showed 228 upregulated (red dot) and 220 down-regulated (blue dot) DEGs in the T2DM group, and the FC (abs) of the most DEGs focused on 1.5–4 (Fig. 2B). The expression alteration of all the DEGs are listed in Additional file 1: Table S1.

### Transcriptional regulatory network construction

**Fig. 3:**
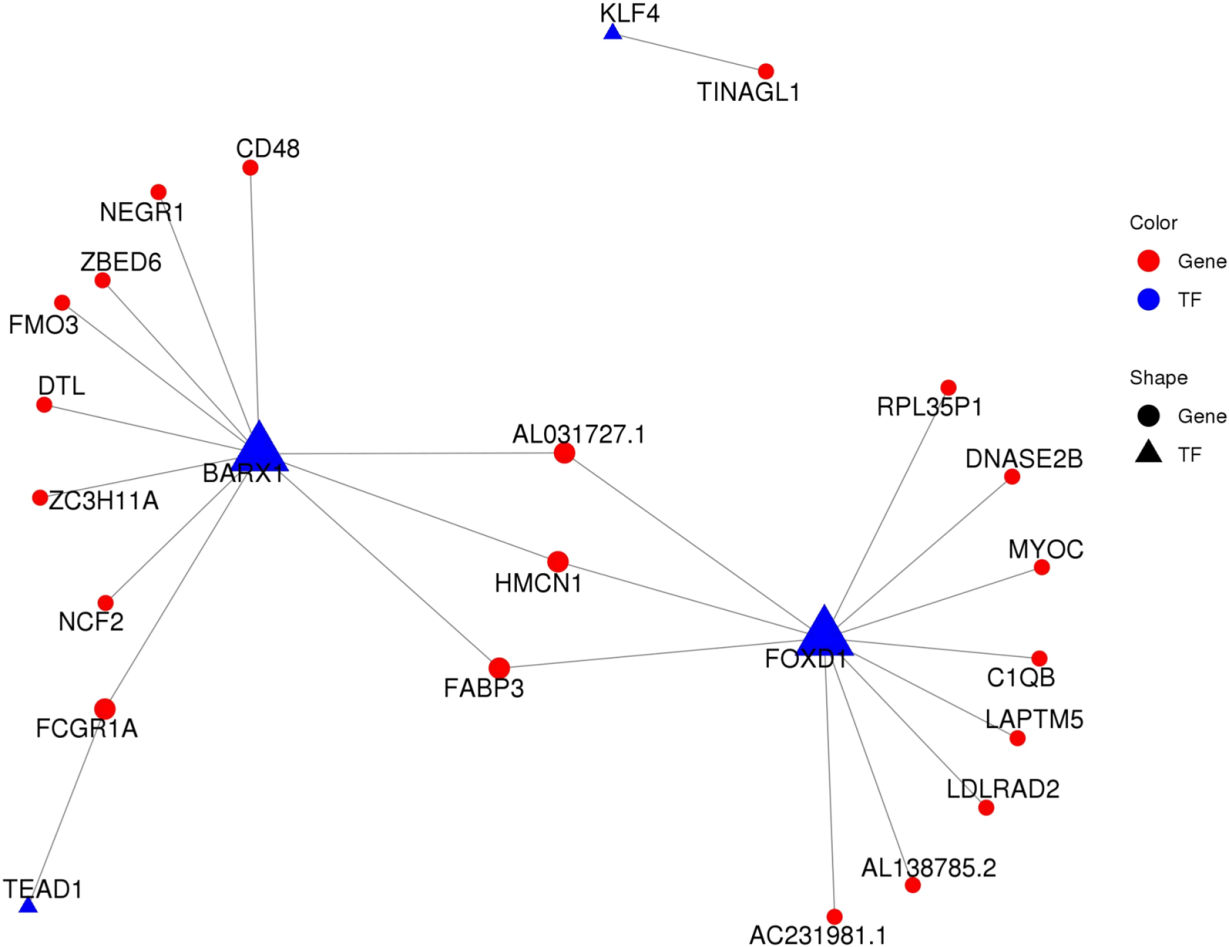
Transcription regulatory network of four transcription factors (blue triangle) and 19 genes (blue circle)

Since the osteogenic differentiation requires the involvement of various TFs, all the 26 TFs were screened out from the 448 DEGs and listed in Table 3. The FC (abs) of top 10 TFs ranged from ~ 4 to ~ 25 (Table 3). A network diagram was built for the TFs and the regulated target genes (Fig. 3). The network comprises 19 genes (blue circle), and four TFs (blue triangle) including KLF4, BARX1, FOXD1, and TEAD1. Both BARX1 and FOXD1 regulated most of the target genes, and also they shared three genes (HMCN1, FABP3, and ALO31727.1) (Fig. 3).

**Table 3:** Expression changes of all transcription factors

	**Gene id**	**TF name**	**Family**	**log2FC**	**log2FC abs**	**FC abs**	***p* Value**	**Up/down**
1	ENSG00000066336	SPI1	ETS	4.66	4.66	25.27	0.008	UP
2	ENSG00000146587	RBAK	zf-C2H2	4.15	4.15	17.70	0.000	UP
3	ENSG00000115956	PLEK	Others	4.02	4.02	16.20	0.015	UP
4	ENSG00000064195	DLX3	Homeobox	− 2.99	2.99	7.92	0.012	DOWN
5	ENSG00000146618	FERD3L	bHLH	− 2.67	2.67	6.38	0.049	DOWN
6	ENSG00000164379	FOXQ1	Fork_head	− 2.35	2.35	5.11	0.005	DOWN
7	ENSG00000131668	BARX1	Homeobox	− 2.16	2.16	4.47	0.001	DOWN
8	ENSG00000069011	PITX1	Homeobox	− 1.88	1.88	3.67	0.004	DOWN
9	ENSG00000168874	ATOH8	bHLH	− 1.84	1.84	3.57	0.001	DOWN
10	ENSG00000257315	ZBED6	zf-BED	1.83	1.83	3.56	0.000	UP
11	ENSG00000162772	ATF3	TF_bZIP	− 1.78	1.78	3.43	0.021	DOWN
12	ENSG00000175592	FOSL1	TF_bZIP	− 1.64	1.64	3.12	0.000	DOWN
13	ENSG00000120738	EGR1	zf-C2H2	− 1.49	1.49	2.81	0.003	DOWN
14	ENSG00000165244	ZNF367	zf-C2H2	− 1.47	1.47	2.76	0.022	DOWN
15	ENSG00000137309	HMGA1	HMGA	− 1.39	1.39	2.63	0.003	DOWN
16	ENSG00000137834	SMAD6	MH1	− 1.28	1.28	2.42	0.016	DOWN
17	ENSG00000067955	CBFB	CBF	− 1.21	1.21	2.31	0.018	DOWN
18	ENSG00000185022	MAFF	TF_bZIP	− 1.21	1.21	2.31	0.007	DOWN
19	ENSG00000172819	RARG	THR-like	− 1.18	1.18	2.27	0.009	DOWN
20	ENSG00000187079	TEAD1	TEA	1.14	1.14	2.21	0.022	UP
21	ENSG00000136826	KLF4	zf-C2H2	− 1.09	1.09	2.13	0.021	DOWN
22	ENSG00000143867	OSR1	zf-C2H2	− 1.07	1.07	2.11	0.049	DOWN
23	ENSG00000108175	ZMIZ1	zf-MIZ	− 1.05	1.05	2.07	0.038	DOWN
24	ENSG00000126368	NR1D1	THR-like	− 1.02	1.02	2.03	0.046	DOWN
25	ENSG00000251493	FOXD1	Fork_head	− 0.97	0.97	1.96	0.042	DOWN
26	ENSG00000177606	JUN	TF_bZIP	− 0.97	0.97	1.96	0.036	DOWN

*FC* fold change, *TF* transcription factor

### Bioinformatics analyses of DEGs

**Fig. 4:**
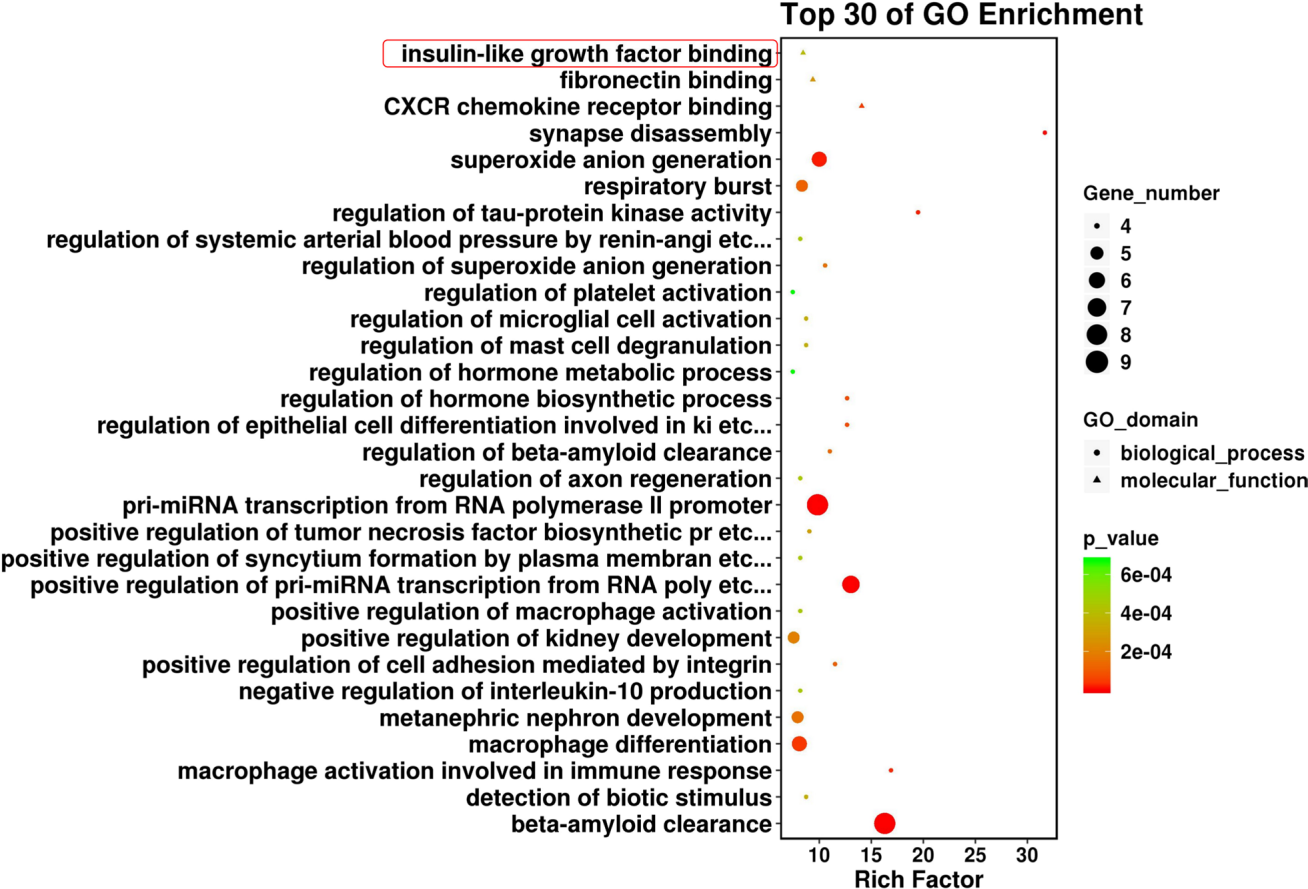
Top 30 GO terms. Circle and triangles represent biological process and molecular function, respectively. The size of the circle/triangle indicates the number of DEGs; the color of the circle indicates the p-value. Diabetes-related term is circled in red

**Fig. 5:**
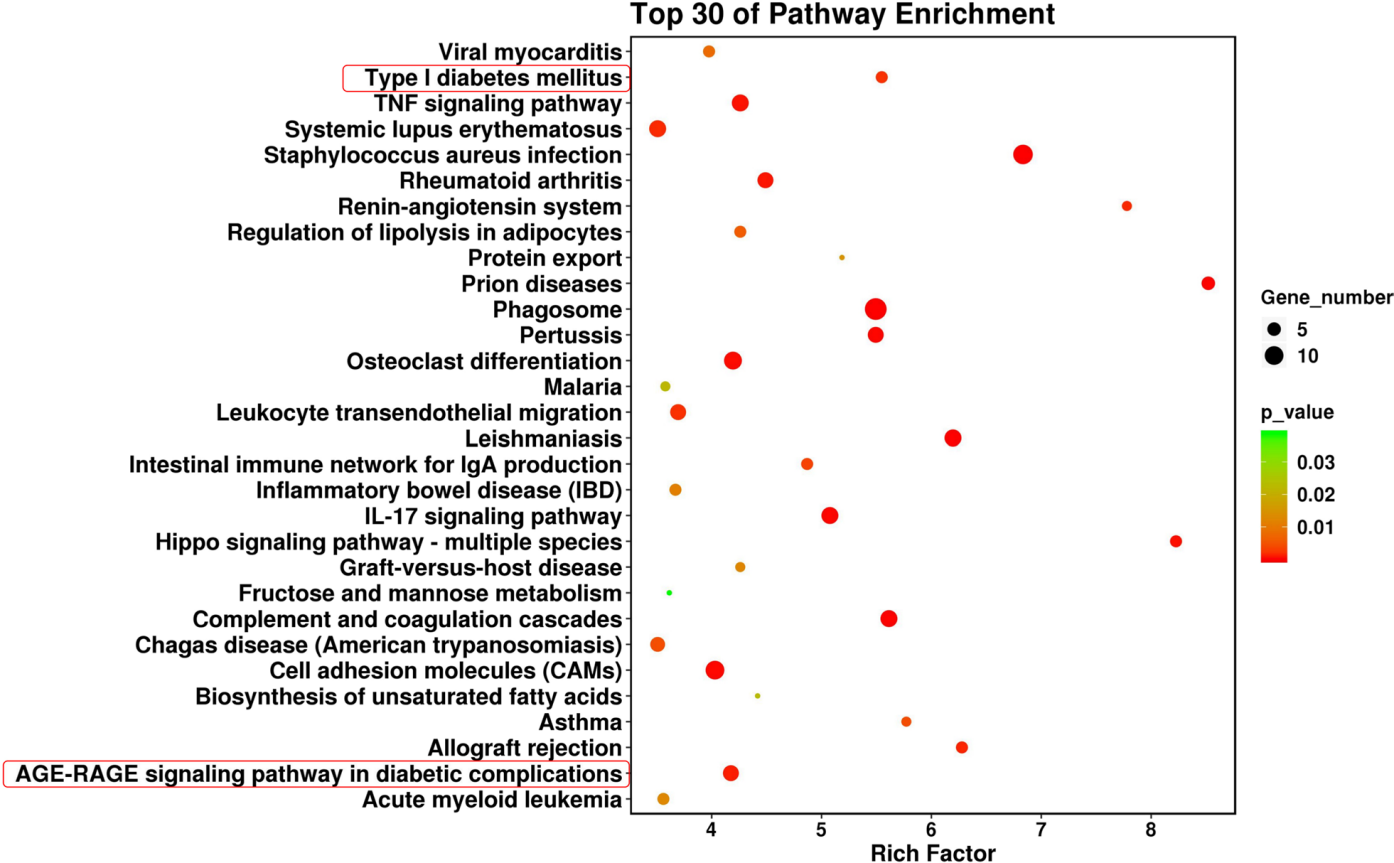
Top 30 KEGG pathways. The size of the circle indicates the number of DEGs and the color of the circle indicates the p-value. Diabetes-related pathways are circled in red

GO analyses were performed to evaluate the potential roles of the DEGs identified from T2DM-BMSC. As shown in Fig. 4, some DEGs were enriched in regulation of hormone biosynthetic process (biological process) and insulin-like growth factor binding (molecular function). The top 30 enriched KEGG pathways are shown in Fig. 5. Some of them were directly related to diabetes, such as type I diabetes mellitus, AGE-RAGE signaling pathway in diabetic complications. The three diabetes related terms and pathways had been circled in red (Figs. 4, 5). Some are related to OP and inflammation including osteoclast differentiation and TNF signaling pathway. All the GO terms and KEGG pathway analyses of DEGs are presented Additional file 2: Table S2.

To find out DEGs involved in OP- and diabetes-related biological processes and pathways, several keywords, “osteo,” “bone,” “diabet,” “insulin,” “glu,” and “glyco,” were used to screen out the relevant GO and KEGG terms. As shown in Table 4, a total of seven TFs (SPI1, SMAD6, CBFB, JUN, FOSL1, RARG, OSR1) (in italic) were enriched in most of the OP-related term and pathways, comparing to the TFs listed in Table 3. Another four TFs (JUN, EGR1, NR1D1, and ATF3) (in italic) were almost involved in all the diabetes-related term and pathways. Additionally, other four genes (FCGR1A, NCF2, MYOC, and FABP3) (in bold) were also enriched in these OP- and diabetes-related biological processes and pathways, comparing to the target genes in the TF network.

**Table 4:** Genes enriched in GO terms and KEGG pathways related to osteoporosis and diabetes

Key words	GO/KEGG ID	GO term/KEGG pathway	Gene UP	Gene DOWN	Enrich factor
Osteo-	GO:0045670	Regulation of osteoclast differentiation	RASSF2, TYROBP, TREM2, PRXL2A	GPR68	3.96
	GO:0030316	Osteoclast differentiation	TYROBP, RASSF2, TREM2, PRXL2A, FCER1G	GPR68	3.07
	GO:0045667	Regulation of osteoblast differentiation	RANBP3L, RASSF2, SFRP2	BMP2, *SMAD6*, VEGFC	2.90
	GO:0001649	Osteoblast differentiation	RANBP3L, VCAN, SFRP2, RASSF2	CYP24A1, **MYOC**, BMP2, *SMAD6*, VEGFC, *CBFB*	2.70
	hsa04380	Osteoclast differentiation	**NCF2**, ACP5, **FCGR1A**, TREM2, *SPI1*, SYK, TYROBP	*JUN*, *FOSL1*	4.19
Bone	GO:0046849	Bone remodeling	ACP5, SYK, RASSF2	GDF5, SYT7	3.23
	GO:0060348	Bone development	RANBP3L, ACP5, INSIG1, SFRP2, TYROBP	**MYOC**, BMP2, GPR68, SCARA3, DCHS1, *RARG*	2.97
	GO:0060349	Bone morphogenesis	SFRP2, ACP5, INSIG1	*RARG*, SCARA3	2.75
	GO:0,030,282	Bone mineralization	FGR, GPM6B	*OSR1*, BMP2	2.09
Diabet-	hsa04940	Type I diabetes mellitus	–	HLA-B, IL12A, HLA-DRB5, HLA-DRB1	5.55
	hsa04933	AGE-RAGE signaling pathway in diabetic complications	AGT, CYBB	*JUN*, *EGR1*, VEGFC, PLCD3, CCND1	4.18
	hsa04930	Type II diabetes mellitus	–	IRS2	1.30
Insulin	GO:0005520	Insulin-like growth factor binding	HTRA3, IGFBP2	IGFBP6, ESM1	8.45
	GO:0061178	Regulation of insulin secretion involved in cellular response to glucose stimulus	FKBP1B, HMGCR	ADCY8, *NR1D1*, GPR68, HLA-DRB1	6.34
	GO:0035773	Insulin secretion involved in cellular response to glucose stimulus	FKBP1B, HMGCR	*NR1D1*, HLA-DRB1, ADCY8, GPR68	5.51
	GO:0032024	Positive regulation of insulin secretion	–	HLA-DRB1, IRS2, ADCY8, GPR68	3.21
	GO:0050796	Regulation of insulin secretion	HMGCR, FKBP1B	*NR1D1*, IRS2, ADCY8, SYT7, GPR68, HLA-DRB1	2.53
	GO:0030073	Insulin secretion	FKBP1B, HMGCR	GPR68, SYT7, ADCY8, HLA-DRB1, IRS2, *NR1D1*	2.17
	GO:0032868	Response to insulin	PLN, **FABP3**, AGT, IGFBP2, FADS1, RARRES2	IRS2, *EGR1*, RAB8A, ADM	2.08
	GO:0032869	Cellular response to insulin stimulus	RARRES2, AGT	RAB8A, IRS2	1.05
	hsa04910	Insulin signaling pathway	FBP1	EIF4E, PTPRF, IRS2	1.74
	hsa04931	Insulin resistance	AGT	PTPRF, IRS2	1.66
	hsa04911	Insulin secretion	–	ADCY8, ADCY4	1.39
Glu-	GO:0061178	Regulation of insulin secretion involved in cellular response to glucose stimulus	FKBP1B, HMGCR	ADCY8, *NR1D1*, GPR68, HLA-DRB1	6.34
	GO:0035773	Insulin secretion involved in cellular response to glucose stimulus	FKBP1B, HMGCR	*NR1D1*, HLA-DRB1, ADCY8, GPR68	5.51
	GO:0071333	Cellular response to glucose stimulus	FKBP1B, HMGCR	GPR68, ADCY8, HLA-DRB1, IRS2, *NR1D1*	2.98
	GO:0001678	Cellular glucose homeostasis	FKBP1B, HMGCR	ADCY8, GPR68, HLA-DRB1, *NR1D1*, IRS2	2.61
	GO:0009749	Response to glucose	FKBP1B, HMGCR	*EGR1*, ADCY8, GPR68, HLA-DRB1, *NR1D1*, IRS2	2.36
	GO:0042593	Glucose homeostasis	FKBP1B, HMGCR	GPR68, *NR1D1*, HLA-DRB1, ADCY8, IRS2	1.66
	GO:0006006	Glucose metabolic process	FBP1, SDS	*ATF3*, IRS2	1.15
	hsa04922	Glucagon signaling pathway	FBP1	–	0.56
Glyco-	hsa00010	Glycolysis/gluconeogenesis	FBP1	–	0.89

Italic genes: transcription factors; bold genes: target genes of transcription factors

### Knockdown of FOXQ1 inhibits the osteogenic differentiation of CON-BMSC

**Fig. 6:**
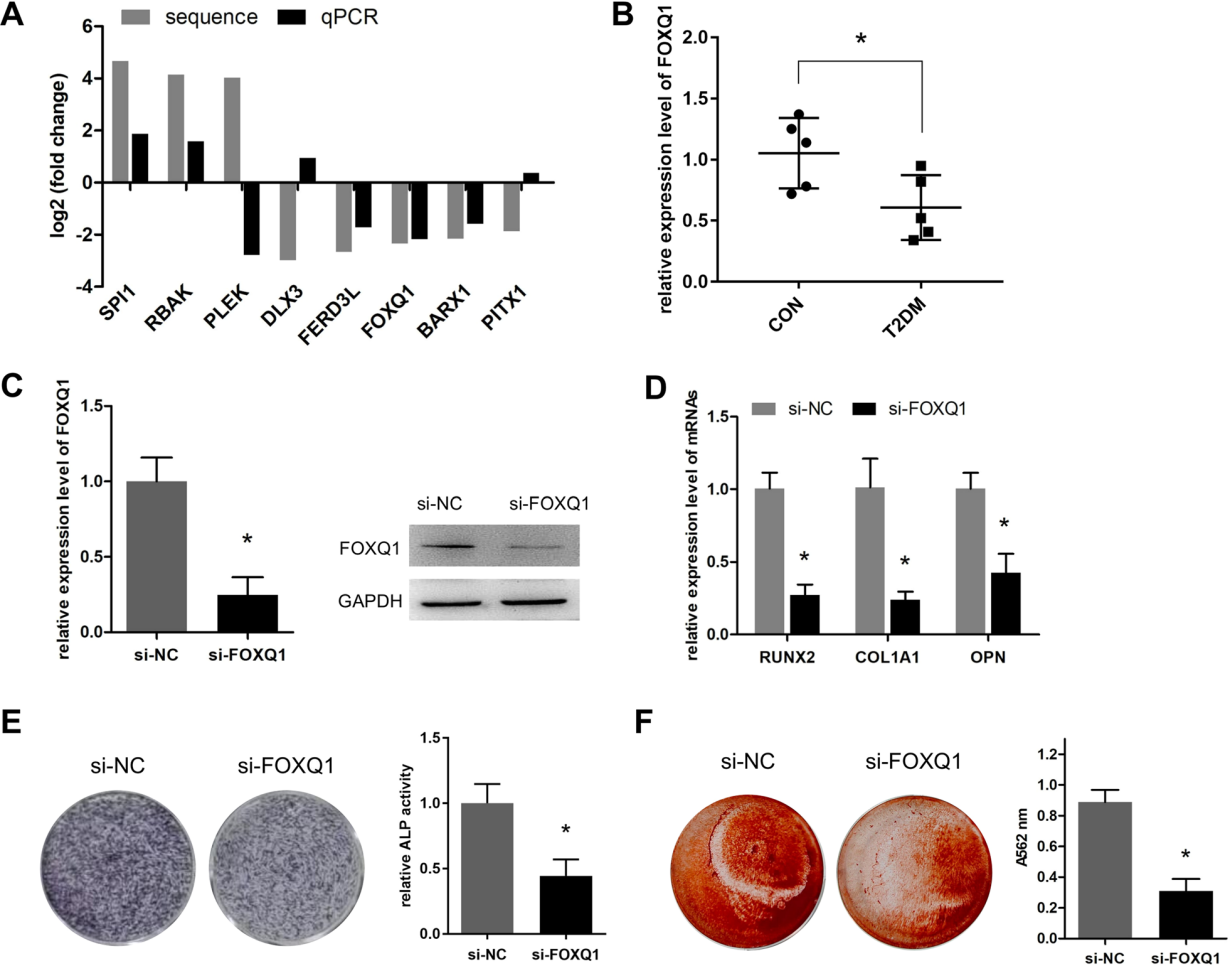
Knockdown of FOXQ1 inhibits the osteogenic differentiation of CON-BMSC. **A** qPCR analysis of the fold change of the top 8 TFs in T2DM-BMSC. **B** The expression level of FOXQ1 in another 10 specimens-derived BMSCs was detected. **C** qPCR and western blot were used to detect the mRNA and protein levels of FOXQ1 after CON-BMSC transfected with siRNAs. **D** qPCR was used to analyze the expression levels of three osteogenic markers in CON-BMSC silenced of FOXQ1. **E** ALP staining (left) and activity detection (right) were performed to detect the role of silencing FOXQ1 on the ALP expression level of CON-BMSC. **F** ARS staining was used to evaluate the role of silencing FOXQ1 on the mineralization level in CON-BMSC

To identify potential genes involved in osteogenic differentiation of BMSC's in T2DM patients, the expression variations of the top 8 TFs were selected for qPCR validation. The result indicated that FOXQ1 had the most significant expression change (Fig. 6A). BMSC from another ten specimens was also isolated and qPCR detection showed that FOXQ1 was significantly decreased in T2DM group (Fig. 6B), hence FOXQ1 was selected for further investigation. Next, we knocked down FOXQ1 in CON-BMSC and confirmed the efficient knockdown of FOXQ1 expression level by qPCR and western blot assays (Fig. 6C). Further, we detected the lower expression levels of RUNX2, COL1A1, and OPN in cells silenced with FOXQ1 (Fig. 6D). In addition, knockdown of FOXQ1 resulted in the significant decrease of ALP activity (Fig. 6E). The ARS staining assay showed that the mineralization level of were reduced significantly, compared to the NC group (Fig. 6F). Collectively, these results revealed that the downregulated FOXQ1 was responsible for the inhibited osteogenic differentiation of T2DM-BMSC.

## Discussion

In the present study, we identified hundreds of DEGs in BMSC from OP patients complicated with T2DM, and annotated the biological roles of the DEGs. More importantly, we found that the TFs and the target genes were involved most of the OP- and diabetes-related biological processes and pathways. And also we identified that the dysregulated FOXQ1 in T2DM-BMSC was responsible for the reduced osteogenic differentiation. Our study provided a potential target to enhance the therapeutic effect of MSC transplantation.

Many studies indicate that the BMD of T1DM patients is reduced, while the BMD of T2DM patients is controversial [18, 19]. Additionally, the hip fracture risk of T1DM is twofold–fivefold of the T2DM patients [20]. Notably, the number of T2DM patients is 8 times more than the T1DM [4], hence T2DM complicated with OP required more attentions. The controversial BMD of T2DM patients might be related to the differences in glycemic control, drug use, and body mass indices (BMI) [21–23]. What’s more, the obesity could suppress the osteogenic differentiation ability of both adipose- and bone marrow-derived MSCs [24, 25]. To uncover the special role of diabetes involved in OP, only the patients with similar BMD, and BMI were considered, and patients with a history of using drugs regulating bone metabolism were excluded.

The gene expression profiles of MSCs from diabetic or OP samples comparing to the healthy controls have been presented in some studies [26, 27], while those from OP complicated with diabetes compared to the healthy or even the OP controls was less investigated. Our study for the first time uncovered 448 DEGs in BMSC-T2DM under the thresholds of FC (abs) > 1.5 and *q* < 0.05. Since the samples in each group were independent, not the parallel, the resulting DEGs was only 18 when FC (abs) > 2 and *q* < 0.05 were used for screen. Therefore, the threshold of FC (abs) was reduced to 1.5 to screen more DEGs. The previous identifies ~ 900 DEGs in diabetic MSCs (vs control MSC) under the thresholds of FC (abs) > 2 and *q* < 0.001 [26]. A much more number of DEGs would be screened out if the filter used equivalent to ours. The major reason for the fewer number of the DEGs in our study was that the control sample was MSCs from OP patients, not the healthy ones. This helped to identify the DEGs involved in diabetic factors relevant OP and rule out those genes involved in spontaneous OP.

The top 30 GO and KEGG enrichment analyses annotated where the genes preferentially enriched in. Diabetes is a chronic disease along with several complications, hence the top 30 terms were involved in various biological processes and pathways. To screen out all the OP- and diabetes-related terms and pathways, several keywords were used and finally a total of 21 terms and pathways were obtained. Interestingly, some of the DEGs enriched in them were the TFs and the target genes. Early growth response protein 1 (EGR-1), belongs to zf-C2H2 family, was enriched in pathway of AGE-RAGE signaling pathway in diabetic complications, and GO terms of response to insulin and response to glucose. It is reported that EGR1-deficient islets fails to maintain the transcriptional network for β-cell compensatory response and EGR1 is regarded as a critical factor in the development of pancreatic islet failure [28]. Notably, silence of EGR1 significantly inhibits the osteogenic differentiation of osteoblast MC3T3 cells and periodontal ligament stem cells [29, 30]. Nuclear receptor subfamily 1 group D member 1 (NR1D1), also named as Rev-erb alpha, is an important transcription factor regulating the function of genes in glucose and lipid metabolism [31]. As we could see, NR1D1 was involved in various insulin and glucose relevant GO terms. Interestingly, recent studies discovered its roles in bone metabolism. Knockdown of NR1D1 in osteoclast precursor cells enhanced osteoclast formation, and expression of osteoclast-associated receptor, while overexpression of NR1D1 in osteoblast precursor attenuated its osteogenic differentiation [32, 33]. The double actions of EGR1 and NR1D1 in diabetes and osteogenesis indicated their potential roles in the development of diabetic OP.

The top 8 TFs were selected for qPCR validation, since most of the 10 TFs, directly involved in OP- and diabetes-related terms and pathways, had a lower FC (abs). As a result, FOXQ1 was found to have the highest verified FC (abs). FOXQ1, also known as hepatic nuclear factor-3 homolog1 (HFH1), belongs to the forkhead box family. It is first confirmed as a regulator of hair follicle development [34]. Later on, it is identified as an oncogene that is over-expressed in various cancers [35]. To date, the role of FOXQ1 in diabetes or osteogenesis is less investigated. Although FOXQ1 was not found to be involved in any OP- and diabetes-related terms or pathways, recent studies showed that its role in osteogenesis or gluconeogenesis is not direct, requiring the involvements of other genes and pathways. For example, FOXQ1 is associated with both adipogenic and osteogenic differentiations of MSCs in an early phase [36]. Overexpression of FOXQ1 promotes the osteogenic differentiation of BMSC via Wnt/β-catenin signaling by binding with ANXA2 [37]. Consistently, our result indicated that silence of FOXQ1 significantly inhibited osteogenic differentiation of CON-BMSC. Notably, the deficiency of hepatic FOXQ1 causes increased blood glucose levels and impaired glucose tolerance, which may contribute to the development of T2DM [38]. FOXQ1 is proved to regulate hepatic gluconeogenesis by the interaction with FOXO1, thereby blocking FOXO1 activity on hepatic gluconeogenesis [38]. The important role of FOXQ1 in diabetes indicated that the reduced FOXQ1 caused the inhibited osteogenesis of BMSC which was one of the vital mechanisms for diabetic OP.

In a word, the present study provided a comprehensive gene expression profile in BMSC derived from diabetic OP and annotated the biological roles of all the DEGs. We also found that the downregulated FOXQ1 was responsible for the development of diabetic OP, and other two TFs, EGR1 and NR1D1, might also play some roles in this pathological process. Our study provided many candidate genes for the special mechanism researches of diabetic OP, which was meaningful for treatment of diabetic OP.

## Supplementary Information

**Additional file 1: Table S1.** The expression alteration of all DEGs in T2DM-BMSC.

**Additional file 2: Table S2.** GO and KEGG analyses of all DEGs.

## Data Availability

The data analyzed during the study are available from the corresponding author on reasonable request.
